# Reducing Stress and Promoting Social Integration of International Medical Students through a Tandem Program: Results of a Prospective-Program Evaluation

**DOI:** 10.3390/ijerph15091959

**Published:** 2018-09-07

**Authors:** Anne Herrmann-Werner, Florian Junne, Felicitas Stuber, Daniel Huhn, Christoph Nikendei, Tanja Seifried-Dübon, Stephan Zipfel, Rebecca Erschens

**Affiliations:** 1 Department of Psychosomatic Medicine and Psychotherapy, Medical University Hospital Tuebingen, University of Tuebingen, 72076 Tuebingen, Germany; anne.herrmann-werner@med.uni-tuebingen.de (A.H.-W.); felicitas.stuber@med.uni-tuebingen.de (F.S.); tanja.seifried@med.uni-tuebingen.de (T.S.-D.); stephan.zipfel@med.uni-tuebingen.de (S.Z.); rebecca.erschens@med.uni-tuebingen.de (R.E.); 2 Centre for Psychosocial Medicine, Department of General Internal Medicine and Psychosomatics, University Hospital Heidelberg, Thibautstraße 2, 69115, Heidelberg, Germany; daniel.huhn@med.uni-heidelberg.de (D.H.); christoph.nikendei@med.uni-heidelberg.de (C.N.)

**Keywords:** tandem project, international medical students, psychological distress

## Abstract

Medical students, and especially international medical students (IMS), have been shown to experience more psychological distress than the general student population in Germany. In order to address these issues, a structured Tandem Program (TP) to reduce stress and foster social integration of IMS has been introduced at the Medical Faculty of Tuebingen. The Tandem Program was evaluated prospectively with perceived stress (PSQ-20) as the main outcome. Secondary outcomes were ‘motives to participate’ in the TP, ‘specific stressors’, and ‘experiences made’ during the program. Stress levels of IMS at the beginning of the program (t_0_) (M = 48.14, SD = 11.95) were higher than those of German participants (M = 39.33, SD = 8.31) (t (67) = −3.66, *p* < 0.001). At the end of the TP (t_1_), stress levels of international students were significantly lower than at t_0_. “Improved ability to work in a team” was seen as one of the most beneficial factors. The results indicate that tandem programs at medical faculties may be a promising curricular intervention towards reducing stress levels, improving integration of international students, and to enhance intercultural and team-oriented competencies in both international and national medical students.

## 1. Introduction

The number of international students (i.e., students seeking education in a foreign country) has considerably increased over the past years, with Europe and North America as the preferred study destinations for the majority of international students [1]. Accounting for up to 15% of all medical students, and with more than 2000 international students taking up medical studies each year, medicine and healthcare sciences are the seventh most popular subjects among international students in Germany [2]. Hence, this subgroup can be seen as a significant population at German medical schools, similar to the situation in the United Kingdom, the United States, and other Western countries [3].

Medical students in general have repeatedly been shown to experience high levels of psychological distress [4,5,6,7,8,9,10]. Those levels are significantly higher when compared with individuals of the same age [9], students of other subjects [7], or with the general population [6]. Additionally, quality of life is lower among medical students compared with the general population [11,12]. When specifically assessed at German universities, the prevalence of stress, depression, and anxiety disorders has also been shown to be higher [13,14,15]. High levels of experienced psychological distress can potentially lead to various problems, such as concentration difficulties, lowered cognitive performance, health problems in general [7], sleep disturbances [10], as well as mental health-related symptoms and disorders, including depression or anxiety [9].

Compared to German medical students (GMS), International Medical Students (IMS) suffer even higher levels of stress [16], reduced quality of life [17], as well as the loss of social contacts in a new environment [18]. Additionally, IMS achieve lower results in preclinical written tests [19], in clinical examinations [20,21,22], as well as state examinations [19], and show an extended duration of study [19,23] as well as higher drop-out rates [18,24]. Erschens et al. [25] showed that 34.6% of variance concerning the amount of subjectively experienced stress within the first semester was accounted for in freshman students from a foreign country of origin. Further distressing factors identified were language and cultural barriers [26,27], lack of social support [28], alienation and homesickness [29,30], financial difficulties [29,31], racial discrimination [30], and lower health-related quality of life [32]. With regards to the issue of sleep disturbance, it is important to note that, for example, sleeplessness must be seen as one important risk factor for suicidal ideations and hence sleeplessness needs to be carefully addressed as another impairing symptom of stress [33].

As IMS represent a vulnerable group of students, many German medical faculties offer general supportive structures (e.g., language classes), and at least one-third of medical schools have established more specific support offers, such as tandem programs (TP) through which a GMS and an IMS are paired in the first semester to form a peer tandem [31,32]. 

Tandem programs, or “buddy programs”, match a more experienced person and a novice in an easily accessible way on a peer-to-peer level to avoid a formally hierarchic dyad in the relationship. In most tandem projects at medical faculties, a resident student, often from a higher semester, takes over the role of a mentor for an international freshman student supporting them according to their needs [31]. Westwood et al. could already show that international students participating in a TP have higher academic achievements and lower dropout rates from the medical curriculum compared to international students not participating in such a matching program [34]. This might be due to the fact that TPs address some of the most prominent problematic factors for IMS, like low social support, cultural differences, and language difficulties [35].

In order to investigate the specific effects of the tandem program of the Medical Faculty of Tuebingen (MFT) on international medical students and on German medical students, the program was evaluated prospectively to investigate the following research questions:

(1) What are the different motives for German and international medical students to participate in the tandem project at baseline (t_0_)?
(2) How does perceived stress differ in German and international tandem participants at the beginning (t_0_) and at the end (t_1_) of the tandem program?
(3) How does perceived stress differ between participants in the tandem program and full cohorts of medical students in similar study phases?
(4) How does the perceived importance of private and study-relevant stressors differ between GMS and IMS?
(5) What are the most helpful experiences of participants (GMS and IMS) during the tandem program?

## 2. Materials and Methods

### 2.1. Study Design

To investigate the research questions outlined above, an exploratory longitudinal design was applied. Participants of the TP (freshman medical students) were invited to take part and fill in questionnaires at two measurement time-points (pre-/post-). Four sequential cohorts of students participating in the tandem project were included in the sample. As an external comparison group, full cohorts of medical students at different stages of their training were analyzed regarding their stress levels. The detailed results of the full-sample investigations were published elsewhere [25]. The main outcome of the study was ‘Perceived Stress’ measured by the Perceived Stress Questionnaire (PSQ-20) [36]. Secondary outcomes of the study were (a) ‘motives to participate in the tandem project’ (at t_0_), (b) ‘specific private and training related stressors’, and (c) ‘experiences made during the tandem program’ (at t_1_). Furthermore, overall satisfaction with the program was assessed with a global item at t_1_.

### 2.2. Intervention—TP for International Medical Students

**Figure 1:**
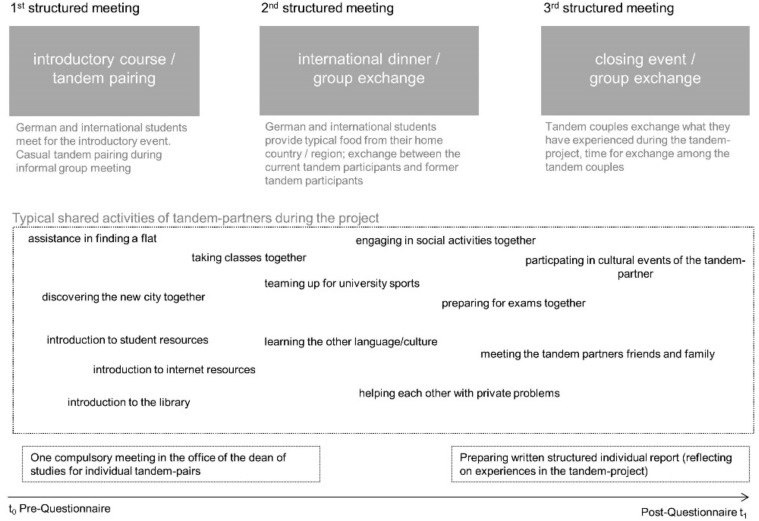
Graphical overview of components of the tandem program (TP).

The TP at the MFT start in the first semester with 10 to 20 international students participating per semester. The class is voluntary and participants are recruited during the so-called “welcome days” prior to the start of the first semester. At the first meeting, the project coordinator presents the content and aims of the project. Thereafter, the tandems consisting of one German and one international student pair themselves through casual encounters. The second meeting mainly focuses on an international ‘potluck’ dinner, with every participant contributing traditional food from their home country. Moreover, former tandem participants from higher semesters (GMS and IMS) are present to report about their experiences when they participated in the project. The third official meeting consists of two parts: first, all tandems report about their tandem experiences within the project and beyond. Afterwards, participants have ample time for personal exchange of experiences. See Figure 1 for further illustrations of the characteristics of the tandem program.

### 2.3. Participants and Measurements

A total of 80 students of 4 subsequent cohorts (freshmen semesters) participating in the TP were asked to participate in the prospective longitudinal evaluation and to fill in questionnaires before the start (t_0_) of the TP and at the end of the first semester (end of the structured/official phase of the TP) (t_1_). Reasons for nonresponse (n = 11) were if students could not come to the official tandem-program meetings where the questionnaires were administered. This was mainly due to sick leave or important duties. The students completed the questionnaires in paper–pencil form. Participants were recruited from 4 sequential cohorts to avoid, e.g., cohort and seasonal effects, as well as to reach sufficient numbers for valid analyses. The structure of the curriculum did not change during the complete recruitment phase, and the educational experience can be assumed to be comparable across the 4 cohorts. The individual numbers of participants for the 4 subcohorts were as follows: subcohort 1: n = 21; subcohort 2: n = 12; subcohort 3: n = 10; subcohort 4: n = 26.

The scope of the applied questionnaire included (a) demographical data, (b) the PSQ-20 [36] (main outcome), and (c) self-generated items focusing on views and experiences regarding specific motives to participate, potential stressors, and experiences during the TP. Category (c) items (self-constructed) were piloted using speaking-out-loud methodology regarding their clarity and practicability. The psychometric properties of the standardized instruments are described in the following.

### 2.4. The PSQ-20

The PSQ-20 was developed to measure self-perceived stress independent of any objective event based on the Lazarus’ model [37,38]. We used the short PSQ-20 version in the German language [36]. The PSQ-20 assessed 4 factors related to perceived stress, “worries”, “tension”, “joy”, and “demands“, with 5 items each. Items were to be rated on a 4-point Likert scale ranging from 1 (“almost never”) to 4 (“usually”). The PSQ-20 sum score, ranging from 0 to 80 points, subsumed all items of the subscales and was widely used in the current literature as a general stress score. Internal consistencies for the PSQ-20 have been reported to vary between Cronbach’s α = 0.80 and 0.86 [36].

### 2.5. Participants’ Motives, Specific Stressors, and Experiences During the TP

Items on the dimensions’ “motives” and “experiences” in the tandem project were created on the basis of qualitative data using the CCSS procedure (collecting, checking, subsuming, and structuring data) [39]. The qualitative data were obtained from written reports of experience from tandem cohorts of previous semesters. Specific private and training-related stressors were also tested in the TP cohort. Participants rated all items across the different dimensions consistently on 5-point Likert scales, with 0 = “strongly disagree”; 1 = “disagree”; 2 = “neither agree or disagree”; 3 = “agree”; and 4 = “strongly agree”. The total number of items for each of the investigated dimensions were: 25 items for the dimension “experiences”, 12 items on “motives”, and 20 items on potential “stressors”. The descriptive results on the investigated dimensions are reported below in rankings listing the top stressors, motives, or most valued experiences according to their mean value, respectively. Such ranking must indeed be interpreted very carefully. For example, it is important to note that a factor ranked at position 1 in a given ranking table, does not mean it is truly important per se, it just means that the mean value of the ratings for this factor were relatively higher than for the other factors. It is also important to note that the distances between a rank 2 and rank 3 may be much different from the distance of the factor ranked 3 to the factor ranked in 4th position.

### 2.6. Ethical Approval and Funding Source

Ethical approval for research into stress levels and associated dimensions in medical students at the MFT was obtained from the ethics committee of the MFT (Nr: 053/2014BO1). All study participants received written study information and gave implicit consent to study participation by filling in the questionnaires. This implicit consent was required by the ethics committee to ensure full anonymity of participants. The study was funded by the Ministry of Science and Education of the German Federal State of Baden-Wuerttemberg via the Competence Center for the Prevention of Mental Health Disorders in Training and Working Contexts (PPAA) Baden-Wuerttemberg.

### 2.7. Statistical Analyses

For summary statistics, mean values (M) and measures of dispersion (standard deviations (SD)) were calculated for sample characteristics (age, gender, response rates) and values of the PSQ-20. Furthermore, mean values and standard deviations were calculated to descriptively compare the perceived relevance of different motives, stressors, and experiences.

Longitudinal within-group differences between GMS and IMS for the main outcome, PSQ-20, were tested using an ANOVA of repeated measures with a post hoc t-test with Bonferoni correction for time points t_0_ and t_1_. If sphericity was violated, the Greenhouse-Geisser correction was applied. The group comparisons between GMS and IMS at the respective cross-sectional time points were calculated using a t-test for independent samples. The significance level of all analyses was set to α = 0.05. All calculations were performed with IBM SPSS Statistics version 24.

## 3. Results

### 3.1. Demographical Data

A total of n = 69 students (33 GMS, 36 IMS) from four sequential cohorts of the TP participated in the prospective evaluation (including participants from two summer-term and two winter-term freshman cohorts). Participants answered the questionnaires at t_0_ and t_1_, respectively. 60% of participants were female, 40% were male. The mean age was 21.9 years (SD = 4.02 years). The frequency of the country of origin of the international students was led by Syria (n = 5), Bulgaria (n = 4), France (n = 3), and Austria (n = 3). Table 1 shows details for sample characteristics and descriptives of the applied standard instrument for both subgroups of GMS and IMS.

### 3.2. Motives to Participate in the TP

The most important motives for participating in the TP among IMS were the hope to promote integration in the study system, to gain more contacts with German students, and to find new friends. Table 2 shows a detailed ranking of motives to participate in the TP from the contrasting perspectives of both subgroups of IMS and GMS.

### 3.3. Differential Perceived Stress in International and German Students

**Figure 2:**
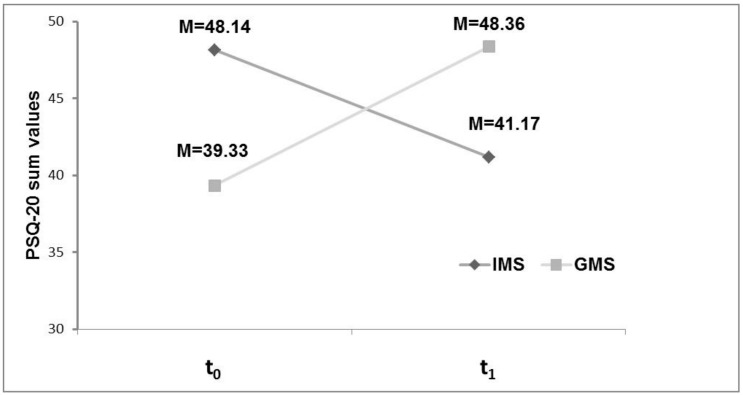
Perceived stress of tandem participants before and after the tandem program. M = mean values for PSQ-20 sum-score values at the two measurement time points, t_0_ and t_1_, for the two groups, IMS and GMS.

With regard to the differential stress levels of the two subgroups, IMS and GMS, involved in the TP, a highly significant interaction between the two factors TP participants and time point with F (1.67) = 22.35, *p* < 0.001, ε^2^ = 0.250 is revealed in the ANOVA with repeated measurements. In the post hoc t-test, the perceived stress of the international TP participants at t_0_ (program start) was significantly higher than at t_1_ (official program end), with t (35) = 2.67, *p* < 0.0125. In contrast, the stress levels of German TP participants at the official end of the TP were higher (t (32) = −4.33, *p* < 0.001). See Table 1 for the descriptive values of the PSQ-20 values and Figure 2 for the values at different times for both subgroups.

**Figure 3:**
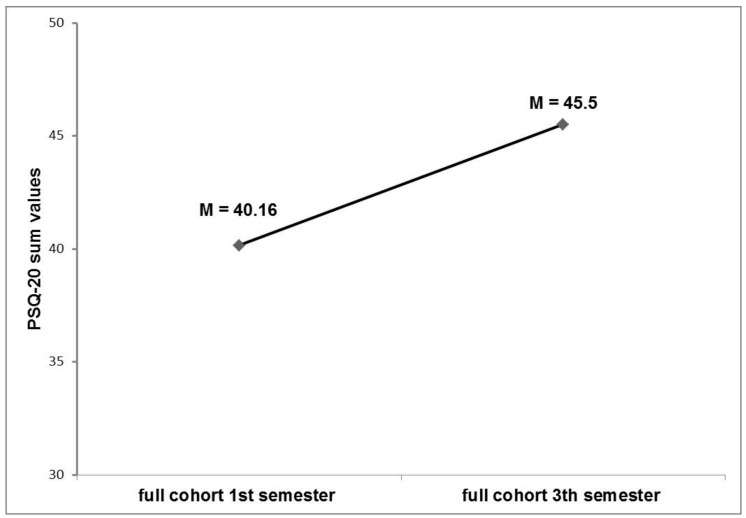
Perceived stress in full cohorts of medical students. M = mean values for PSQ-20 sum-score values of two full cohorts of medical students at comparable study levels to the measurement time points of the prospective evaluation of the tandem program (C1: freshmen medical students, n = 148; C2: medical students at later study stages (third semester), n = 143).

In the cross-sectional comparison at t_0_, IMS in the TP showed significantly higher PSQ-20 values (M = 48.14, SD = 11.95) than GMS (M = 39.33, SD = 8.31) (t (67) = −3.66, *p* < 0.001). Inversely, at time-point t_1_, German students showed significantly higher PSQ-20 sum values (M = 48.36, SD = 13.19) than international students (M = 41.17, SD = 11.03) (t (67) = 2.49, *p* < 0.05). See Figure 2 for a graphical illustration of these comparisons. Figure 3, in contrast, shows the values for PSQ-20 for the total cohorts of medical students at the first and the third semester, illustrating that stress level seems to increase in first semesters (see Erschens et al. [25] for further details).

### 3.4. Most Important Specific Stressors from the View of IMS vs. GMS

The five specific stressors with the highest ratings for importance from the contrasting views of the two investigated subgroups of IMS and GMS are shown in Table 3.

At t_1_ (end of the program), the study participants were asked to rate their general opinion regarding the tandem program in a global item regarding overall satisfaction on a categorial school grade scale ranging from “very good” (= 1) to “insufficient” (= 5). In the subgroup of GMS 24.2% rated the program in retrospect overall as “very good” and 54.5% as “good”. In the subgroup of IMS 44.4% rated the program overall as “very good” and 27.8% as “good”.

Furthermore, at t_1_ (at the end of the program) the participants rated a comprehensive set of potential specific experiences according to their perceived importance (degree of agreement with a given statement) on a 5-point Likert scale. Table 4 shows the 10 most important experiences made in the program for each group.

### 3.5. Experiences and Views of IMS and GMS during the Tandem Project

The five most important experiences and views with the highest ratings of importance from the contrasting views of the two investigated subgroups of IMS and GMS are shown in Table 4.

## 4. Discussion

To the best of the authors’ knowledge, this is the first study to prospectively evaluate a tandem curriculum for international medical students with regards to motives to participate, perceived stress, specific stressors, and experiences made during the program. The results show that participants were overall satisfied with the program, with high proportions (of both subgroups) rating the program as “very good” or “good”.

The results further show that stress levels in IMS are very high at the beginning of the program (beginning of medical studies), and that perceived stress appears to be lower in IMS at the end of the program. Perceived stress in German participants of the tandem project, on the contrary, seems to be higher at the end of the program compared to the beginning. With regards to the second research question of this study, it can therefore be summarized that stress levels show a mixed picture, with lower stress levels in IMS at the end of the program but higher perceived stress in GMS after participation in the TP/after completion of the first semester of medical studies. This is the case, albeit both groups have the same curriculum and need to pass the same exams. The interesting finding is that the stress levels of IMS being nevertheless lower at the end of the first semester compared to GMS may be explained by the potentially different mindsets of the two groups, especially when starting medical studies. IMS may have had relatively better experiences during the first semester compared to their (maybe rather negative or anxious) expectations before starting to study medicine (since they worried, e.g., with regards to language barriers). The relief of IMS to have mastered the first semester might have been more intense in IMS than in GMS. The latter might have less expected to struggle or to have difficulties during the first semester. GMS might also have expected to perform better and get higher grades in exams, whereas IMS may have been more focused on passing all summative examinations, no matter what grade. Anecdotal evidence that the authors have from personal interactions with participants of both groups point in these directions. This effect can be seen as based on an aspiration vs. result mismatch (in GMS) or match/overfulfillment (in IMS) may explain more intense relief after the first semester (and hence lower stress levels) in IMS, and relatively more “grief” (and hence potentially higher stress levels) in GMS.

To answer the third research question of this study, the values of the investigated tandem subgroup of GMS here were compared with two full cohorts of medical students at similar study phases (see Erschens and colleagues [25]). Here, the GMS subgroup of tandem participants resembles the higher rates of perceived stress of the full cohorts of medical students at later study time points. Hence, the values may not be interpreted as solely explainable by participation in the tandem program, but may be in line with the overall trend of perceived stress in medical students in preclinical stages. This is in line with several previous findings in the literature: The studies of Aktekin et al. [5] and Guthrie et al. [40] showed an increase of perceived stress in medical students from the beginning of their studies. Aboalshamat et al. [9], Ludwig et al. [41], and Waqas et al. [10], on the other hand, noticed an increase of perceived stress in the third semester. This increase in the preclinical stage could be related to study-related stress like exams. 

Despite the fact that GMS stress scores seem to be in line with a general picture of the full cohorts of students, one might contemplate that participating in the tandem project might nevertheless be a stressor for German students since they might be supportive for their international tandem partner, but might not benefit to the same extent from their international buddy. In a casually paired tandem, the relationship between the GMS and the IMS might not have developed well, or the integration of the IMS was a very challenging task, like 36% of the medical faculties stated in Huhn et al. [31]. However, looking at the results regarding the most important specific stressors within the program, none of the stressors with the highest rankings within the GMS subgroup (see Table 3) is related directly to the relationship with the tandem buddy. In contrast to the IMS subgroup, the GMS rated the factor “time management” relatively high. The result that “time management” was a stress-related factor in medical students is in line with earlier studies regarding medical students in the United Kingdom who did not participate in a TP [42]. This aspect might support the idea that the increase of the perceived stress in GMS is not related to the TP. Another point of discussion could be that the additional (perceived) duties within the tandem project are part of the time-management issues as seen by GMS.

With respect to the latter factor, one could discuss whether the additional (perceived) duties within the tandem project are part of the time-management issues as seen by GMS. To answer the fourth research question of this study more generally, the stressors that were ranked as the most important from the views of both subgroups were predominantly all study-related with, for example, qualitative (content-related) and quantitative (study load-related) requirements. This result has been shown by Radcliffe and Lester as well: they conducted semistructured interviews with Year 5 students, and also identified study-related aspects, with work pressure and gaining expertise in the medical field as two of the most burdensome factors [43]. Nevertheless, it is important to keep the relationship between private stressors and study-related stressors in mind. Misra et al. showed that a huge amount of life stress and little social support predicted higher levels of academic stress [28]. In the case of IMS, “financial worries” were among the five most important stressors as the single stressor that was not directly related to the study context, but rather related to the personal or private circumstances of the participants. The issue of financial worries was addressed previously in a study by Huhn et al. [44]. Given the results of this study one can see a difficult financial situation of international students as contributing to a vicious circle, being forced to work to earn money, thus having less time and energy to study or take German language classes, and subsequently eventually scoring even lower in examinations, which, in turn, increases stress rates. In a systematic survey of German faculties by Huhn and colleagues [19] about the perceived problems of international students, by far the most frequently reported problem was the lack of language skills of the international students at the beginning of their studies. Interestingly, the potential lack of language skills at IMS in self-assessment in this study did not appear among the five most stressful stressors.

With regards to the motives for participating in the TP, IMS rated the reason “to be better integrated into existing systems” as the most important factor, whereas GMS rated the “hope that the program will make it easier for international students to enter university” as their most important motive to subscribe. The second most important motive for GMS was found to be “I want to help international students”. The latter two motives may be seen as a rather altruistic, in contrast to more self-oriented motives found in the IMS group. The only self-oriented motive of GMS in the top-five ranking was “I want to learn more about foreign cultures”. Hence, one might conclude with regards to the first research question that, in the beginning, GMS showed rather altruistic motives, whereas IMS were seeking help in the tandem program based on more self-oriented motives. However, we have to keep in mind that TP was kind of an optional subject, and GMS as well as IMS could acquire ECTS for their participation.

One interesting result was that both statements regarding the experiences made in the TP, which received the highest ratings, was the global item “I would participate in the TP again” and “I understood well with my tandem partner”. Hence, one might at least question the above-discussed issue of imbalanced benefits between GMS and IMS in the TP if GMS so clearly state that they would participate again in the TP.

From a more general perspective, the findings of this study seem to strongly support the attempt to offer structured tandem programs to international freshmen medical students at medical schools. Not only were the general ratings from both subgroups (IMS and GMS) clearly positive, but also social skills such as “…ability to work in a team” (rank 9 in IMS, see Table 4) or “…improvement of social skills” (rank 9 in GMS, see Table 4) were perceived as improved by participants. Such positive effects do not only foster the skills and the related self-confidence of participants, but can also be seen as essential skills for future physicians. The same may apply to the intercultural skills that were appreciated by the TP participants (rank 6 in GMS and rank 10 in IMS, see Table 4). Intercultural skills, in particular, are becoming more and more important in a globalizing world and in the medical field [45].

Interventions such as the tandem program may stipulate personal growth by creating opportunities to improve social, reflective, and communicative skills. Furthermore, in the case of a tandem program for IMS, there may be the learning opportunity of how to identify with a “helping role” (for GMS) and IMS (in parts) experiencing a potentially “dependent” role. While the relationship of a tandem pair unfolds, these two roles might become not so clearly distributed and students might be able to reflect how interactions of human beings are rather interdependent and imply the obligation to act responsibly to serve the personal growth of oneself and the tandem partner alike.

Hence, besides potential positive effects on perceived stress and social integration of international students at medical schools, tandem projects or similar programs may serve as an instrument to stipulate personal growth in students in dimensions that might serve as resources during their further medical studies and indeed as future physicians [46]. However, tandem programs are indeed not limited to the context of medical schools but are easily transferable and certainly effective in totally different areas of the academic realm as well.

### Limitations

The interpretation of results is limited due several limitations: First, we only had the external comparison group for the general trend in stress levels of medical students. A randomized and directly controlled design was not feasible in the study context due to the fact that no student could be excluded from an official curricular offer by the medical faculty when they wanted to actively participate. A waiting group design as an alternative was not applied because the TP officially runs in the first semester from the first week of study to the last week of the first semester. Therefore, the external comparison was chosen to consist of full cohorts of medical students as a proxy of a fully sufficient control group. Second, the design was exploratory and not hypothesis-driven in nature. There may be many further factors that influence the individual stress-levels of medical students that were not adequately included in this study. Hence, none of the presented results should be understood as implying totality or causality. Third, the group of IMS were very heterogeneous with regards to their country of origin and hence may face very different issues during the integration process. However, subgroup analysis regarding country of origin was hindered in this study by insufficient subgroup sizes. Further limitations of the study lie in the characteristics that all investigated dimensions relied on self-report questionnaires. None of the outcome parameters, such as “improved social skills”, were assessed objectively, for example, by appropriate tests or observations. However, since the study mainly focused on motives, perceived stress, factors of distress, and experiences made, the subjective nature of the assessment was decided to be adequate. Future studies, however, may benefit from objectively assessing the subjective improvements reported here or could compare perceived stress levels between different optional subjects in medical students in the same semester.

## 5. Conclusions

In conclusion, this study showed that a structured tandem program might be a promising curricular instrument to reduce mental distress in international students who, in turn, might aid to stipulate the social integration of students from foreign countries. Furthermore, German tandem participants also rated the program very positively on average and made experiences that may potentially contribute to their personal growth, including social and intercultural skills that may be needed to fulfil their aspirations as future physicians.

## Figures and Tables

**Table 1:** Sample characteristics and descriptives of applied standard instruments.

			International Medical Students (IMS)	German Medical Students (GMS)	All
Dimensions	response rate		36/40	33/40	69/80
	Gender ^1^	f (%, n)	50.0%, 17	69.7%, 23	59.7%, 40
		m (%, n)	50.0%, 17	30.3%, 10	40.3%, 27
	age (M,R)		21, 27, 18–28	22, 66, 18–39	21, 89, 18–39
Perceived Stress (PSQ-20)	T_0_ M (SD), n		48.14 (11.52), 36	39.33 (7.95), 33	43.93 (10.85), 69
	T_1_ M (SD), n		41.17 (10.70), 36	48.36 (13.19), 33	44.61 (12.45), 69

Note. M = mean, R = range, SD = standard deviation, PSQ = Perceived Stress Questionnaire, T_0_ = measurement time point before the intervention, T_1_ = measurement time point after the intervention; ^1^ no data provided by two tandem participants, therefore n = 67.

**Table 2:** Ranking of most important motives for participating in the TP.

**International Medical Students**	
**(Rank) Item**	M (SD)
(1) “I think the project will help to be better integrated into existing systems”.	3.42 (0.94)
(2) “I’m looking for more contacts with German students”.	3.27 (0.76)
(3) “I want to make new friends”.	3.18 (0.94)
(4) “I would like to improve my German language skills”.	3.18 (0.92)
(5) “I am seeking cultural exchange”.	3.15 (0.94)
Items that received lower ratings included factors such as: “getting curricular credit points”, “seeking help with private problems”, “making new friends with other international students”, “seeking help with study content”.	
**German Medical Students**	
**(Rank) Item**	M (SD)
(1) “I hope that this project will make it easier for international students to enter university”.	3.61 (0.61)
(2) “I want to help the international students”.	3.56 (0.61)
(3) “I want to learn more about foreign cultures, languages and habits”.	3.56 (0.72)
(4) “I have great respect for the courage of international students to go to a foreign country to study medicine”.	3.52 (0.62)
(5) “I want to contribute to the integration of international students”.	3.42 (0.75)
Items that received lower ratings included factors such as: “getting curricular credit points”, “getting better in structuring and organizing my studies”, “I want to take responsibility for another person”.	

Note. Participants rated all items on a 5-point Likert scale, with 0 = “strongly disagree”; 1 = “disagree”; 2 = “neither agree or disagree”; 3 = “agree”; and 4 = “strongly agree.

**Table 3:** Most important specific stressors from the view of IMS vs. GMS.

**IMS**	
**(Rank) Item ^1^**	M ^2^ (SD)
(1) study requirements (qualitative)	2.25 (0.94)
(2) study load (quantitative)	2.22 (1.17)
(3) selection-performance pressure	2.17 (1.07)
(4) financial worries	2.12 (1.42)
(5) beginning of medical training at large	1.79 (1.10)
**GMS**	
**(Rank) Item**	M (SD)
(1) beginning of medical training at large	2.29 (0.81)
(2) study-load (quantitative)	2.17 (0.82)
(3) selection-performance pressure	2.04 (1.12)
(4) time management/working style	1.87 (0.961)
(5) study requirements (qualitative)	1.83 (0.872)
Items that received lower ratings by both subgroups included factors such as: “the relationship with my fellow students”; “the relationships with professors and university staff”; “missing support from services of the medical school”; “conflicts in private relationships”.	

Note. ^1^ For an overview of the complete list of stressors offered see [25]. ^2^ Participants rated all items on a 5-point Likert scale, with 0 = “strongly disagree”; 1 = “disagree”; 2 = “neither agree or disagree”; 3 = “agree”; and 4 = “strongly agree”.

**Table 4:** Most important experiences and views of IMS and GMS regarding participation in the TP.

**International Medical Students (IMS)**	
**[Rank]** item	M (SD)
(1) “I *understood well* with my tandem *partner*”	3.48 (0.99)
(2) “I would *participate* in the tandem project *again*”	3.37 (1.19)
(3) “the *situation of international* students has *improved* as a result of the project”	3.23 (0.97)
(4) “through the tandem project, I *made new friends*”	3.13 (1.11)
(5) “I experienced my buddy as my *supportive partner*”	3.00 (1.34)
(6) “The tandem project has *fulfilled my expectations*”	2.83 (1.18)
(7) “the tandem project was a balanced “*giving-and-taking* “for me”	2.63 (1.07)
(8) “Through the tandem project I learned to *understand cultural differences* better”	2.57 (1.19)
(9) “The tandem project has enabled me to become *more able to work in a team*”	2.47 (1.01)
(10) “the tandem project has contributed to the **reduction of prejudices**”	2.40 (1.13)
**German Medical Students (GMS)**	
**[Rank]** item	M (SD)
(1) “I would *participate* in the tandem project *again*”	3.57 (0.77)
(2) “I *understood well* with my tandem *partner*”	3.56 (0.73)
(3) “the *situation of international* students has *improved* as a result of the project”	3.43 (0.63)
(4) “through the tandem project, I *made new friends*.”	3.20 (1.00)
(5) “The tandem project has *fulfilled my expectations*”	2.83 (0.99)
(6) “Through the tandem project I learned to *understand cultural differences* better”	2.73 (0.87)
(7) “the tandem project was a balanced “*giving-and-taking* “for me”	2.70 (1.24)
(8) “I experienced my tandem partner as my *supportive partner*”	2.37 (1.25)
(9) “Through the tandem project I was able to *improve my social skills*”	2.23 (0.86)
(10) “the tandem project *improved* my general *Well-being*	2.20 (0.97)
Items that received lower ratings by both sub-groups included factors such as: “the tandem-program was a burden for me”; “through the tandem-program my empathy for others has improved”, “my role within the tandem program was not clear to me”	

Note. n = 30 Participants rated all items on a 5-point Likert scale with 0 = “strongly disagree”; 1 = “disagree”; 2 = “neither agree or disagree”; 3 = “agree” and 4 = “strongly agree”.
